# Design Fluency in Children with ADHD and Comorbid Disorders

**DOI:** 10.3390/brainsci10030172

**Published:** 2020-03-17

**Authors:** Anaïs Fournier, Bruno Gauthier, Marie-Claude Guay, Véronique Parent

**Affiliations:** 1 Department of Psychology, University of Sherbrooke, Longueuil, QC J4K 0A8, Canada; Anais.Fournier@USherbrooke.ca (A.F.); Veronique.Parent3@USherbrooke.ca (V.P.); 2 Department of Psychology, University of Montreal, Laval Campus, Laval, QC H7N 0B6, Canada; 3 Department of Psychology, University of Quebec in Montreal, Montreal, QC H3C 3P8, Canada; guay.marie-claude@uqam.ca

**Keywords:** executive function, developmental disabilities, attention deficit and disruptive behavior disorders, oppositional defiant disorder, anxiety disorders

## Abstract

Background: Attention deficit/hyperactivity disorder (ADHD) is often associated with frontal executive impairment in children. Oppositional defiant disorder (ODD) and anxiety disorders (AD) frequently accompany ADHD, but the impact of these comorbid disorders on cognition remains elusive. The five-point test (FPT), a design fluency task, has been shown to be sensitive to neurological damage, specifically to frontal lobe lesions in patients with brain injuries. The purpose of this study was to compare the performances of neurotypical children with that of children with ADHD, ADHD-ODD, and ADHD-AD on the FPT in order to examine whether these groups could be distinguished from one another based on their cognitive profile. Methods: A total of 111 children aged 8 to 11 years old participated in the study. Six measures from the FPT were used to characterize their performance. Results: Statistically significant differences between groups were observed for five of the six FPT measures. Essentially, children with ADHD-ODD made more repeated designs than the three other groups (control *p* > 0.001, ADHD *p* = 0.008, ADHD-AD *p* = 0.008), while children with ADHD-AD produced fewer total and correct designs than the control and ADHD groups (*p* = 0.009). Conclusions: This suggests that comorbidities have an additive impact on the cognitive profile of children with ADHD. Design fluency may be a sensitive measure for capturing the subtle cognitive deficits that are likely to be involved in these disorders.

## 1. Introduction

Attention deficit/hyperactivity disorder (ADHD) is characterized by persistent inattention and hyperactivity/impulsivity and is one of the most prevalent neurodevelopmental disorders [1]. The disorder is also associated with executive function impairment, such as poor inhibition, working memory, cognitive flexibility, or planning [2,3,4,5,6,7]. However, increasing evidence indicates cognitive heterogeneity in ADHD [6,8,9,10,11,12]. One possible explanation is the presence of confounding factors such as comorbid disorders, which are not always controlled in ADHD studies [13]. Poor sensitivity of executive function assessment tools may also account for part of the heterogeneity. In this study, we investigated the utility of design fluency for comparing neurotypical children to children with ADHD alone and with its two most common comorbid problems: oppositional defiant disorder (ADHD-ODD) and anxiety disorders (ADHD-AD).

Design fluency was initially developed by Jones-Gotman and Milner [14] as the nonverbal counterpart of verbal fluency [15]. It involves the coordination of multiple executive functions in the visuospatial domain including generativity, self-monitoring, and planning [16,17]. The task requires drawing as many different non-symbolic designs as possible in a limited amount of time by connecting dots with straight lines in each square of a matrix. Standard measures of design fluency include the total or correct number of designs, the total or ratio number of repeated designs (repetition errors), and the number of strategies [18], the latter supposedly helping quantify the use of a stratagem to promote design production while avoiding errors [19]. The design fluency five-point test (FPT [20]) was shown to be sensitive to frontal lobe damage in adults [21]: patients with neurological conditions produced fewer designs and more repeated designs than patients with psychiatric disorders, and repeated designs could also distinguish between anterior and posterior lesions and showed a tendency to distinguish between right and left anterior lesions—patients with right frontal lobe damage producing more repetitions.

Decreased frontal/prefrontal activity has been reported in individuals with ADHD (e.g., [22,23,24]) as well as prefrontal cortex dysfunction in youth with ADHD [25,26,27]. Imaging studies have shown cortical volume reductions in diverse regions in children with ADHD, including subcortical regions (i.e., bilateral amygdala, accumbens, and hippocampus) [28], but also the right frontal and prefrontal regions [29,30]. Since executive functions are most affected in ADHD and associated with the prefrontal cortex [31] and visuospatial abilities with the right side of the prefrontal cortex [32], the FPT could be useful in documenting executive deficits in children with ADHD.

To our knowledge, only four studies have tested children with ADHD using design fluency. In the first one, Loge, Staton, and Beatty [33] compared 20 children with ADHD (17 boys) with 20 children of a control group (17 boys) on a design fluency test that required producing as many unique designs using four lines. Both groups produced a similar number of unique designs. However, children with ADHD broke more rules (number of designs containing more or fewer than four lines), which the authors attributed to a possible orbitofrontal dysfunction. Repeated designs and strategies were not considered. In their longitudinal study, Robinson and Tripp [34] compared the performance of 55 children with ADHD and 55 children from a control group on a design fluency task using five scores: total number of designs, number of correct designs, number of repetition errors, number of nonconformity errors (i.e., other than repetition), and total number of errors. Strategies were not considered. Children with ADHD produced significantly less correct designs and more nonconformity errors than the control group, with no difference on other scores. The authors hypothesized a deficit in creativity rather than in self-regulation. A study by Vélez-van-Meerbeke et al. [35] included 119 children with ADHD and 85 children from a control group aged 6 to 13 years old. The ADHD group produced significantly fewer designs than the control group. Once again, strategies were not considered. More recently, Gauthier, Parent, and Lageix [36] observed no difference on the total number of designs produced by 34 children with ADHD and 37 children without ADHD aged 8–11 years old.

Despite the potential interest of design fluency in ADHD, there are limited and rather incomplete and inconsistent data. Part of the discrepancy may stem from the lack of consideration for the frequent comorbidities associated with ADHD in childhood. Between 30% and 66% of children with ADHD also present with comorbid ODD [37,38,39] and 40% have anxiety problems [40,41]. Research suggests that ADHD, ODD, and anxiety, although they differ in terms of precise connectivity patterns, share similar neurological mechanisms related to the frontal and prefrontal cortices and subcortical regions.

In children, ODD is believed to be associated with abnormalities of the dorsolateral prefrontal cortex and the cerebellum [42,43,44,45] as well as of the amygdala, anterior cingulate cortex, and insular cortex [42,43,46]. The amygdala, striatum, insular cortex, and frontal gyrus are structures that are involved in emotional processes [47,48], reinforcement [49,50], and introspection [51,52]. This would explain the emotional and behavioral regulation difficulties, the altered response to reinforcement [53,54,55], and the inhibition deficit often observed in children with ODD [56,57,58]. Increasing evidence also suggests that the ADHD-ODD comorbidity is associated with motivational and inhibitory dysfunctions (e.g., [59,60]).

Similarly, several studies support the involvement of the prefrontal cortex in anxiety (e.g., [61,62]). Post-traumatic stress disorder is associated with decreased activity of the dorsolateral prefrontal cortex [63], while generalized anxiety disorder is associated with decreased activation of the dorsolateral and dorsomedial prefrontal cortex [64]. Recently, a meta-analysis of neuroimaging studies aimed to identify the neural substrates that are common and specific to different anxiety disorders [65]. Results indicate that common neural networks are involved in various anxiety disorders, specifically those connecting the limbic system (amygdala and insular cortex) to regions responsible for self-regulation (medial prefrontal cortex and anterior cingulate rostral cortex). It also appears, however, that the neurobiological underpinnings of anxiety vary from one disorder to another. The presence of anxiety may reduce inhibition deficits, but negatively impact on attention and working memory in individuals with ADHD [41,66,67]. However, a recent meta-analysis concluded that AD has a protective role for attention in older children and for working memory in boys with ADHD [68].

No study to date has used design fluency to compare the performance of children with ADHD alone and comorbid ADHD. In this study, we used six measures of the FPT for characterizing ADHD with or without comorbidity. Considering the study by Lee et al. [21] and the neural substrates involved in ADHD, ODD, and AD, we predicted that children with ADHD would produce more repeated designs than the control group. Moreover, considering the association of both ODD and AD with the prefrontal and limbic systems, we predicted a higher number of repeated designs and a lower number of designs in the comorbid groups. Since no study so far has focused on strategy production in ADHD, we examined this measure in an exploratory fashion.

## 2. Materials and Methods

### 2.1. Participants

A total of 111 children aged 8 to 11 years (72 boys; mean age = 9.73 ± 0.99 years) participated in the study. The control group (without ADHD) consisted of 34 children (16 boys; mean age = 10.03 ± 1.06 years) from regular schools. The clinical groups consisted of 77 children (56 boys; mean age = 9.58 ± 0.96 years) divided into three groups (see Table 1): ADHD (ADHD), ADHD with associated ODD (ADHD-ODD), and ADHD with associated AD (ADHD-AD). Children with ADHD were diagnosed with combined presentation. Although children with ADHD and comorbid ODD often have comorbid AD, children in our sample either had ODD or AD. The diagnoses were made on the basis of a standardized biopsychosocial and psychiatric assessment (see Procedure section). Children with known intellectual delay or neurological disorder (e.g., epilepsy) were excluded from the study. To control for the daily use of medication for ADHD in the clinical groups, 43 children from the clinical groups were asked not to take their medication on the day of the cognitive assessment. Children taking Strattera^®^ were excluded from the study since atomoxetine cannot be eliminated from the body fast enough.

### 2.2. Procedure

Children on the waiting list of the specialized ADHD clinic in Montreal Rivière-des-Prairies Hospital went through a biopsychosocial and psychiatric standardized evaluation involving three steps to identify the presence of ADHD and a comorbid disorder: (a) a developmental and behavioral assessment with questionnaires completed by parents and teachers, (b) a cognitive assessment (of attention and executive functions), and (c) a diagnostic evaluation of mental disorders based on the Diagnostic and statistical manual of mental disorders, fourth edition (DSM-IV) [69] and conducted by a child psychiatrist. The cognitive assessment lasted approximately 90 minutes and included the FPT. After the cognitive assessment, the study was presented by the psychologist who evaluated the child to the parent(s) and the child, who signed the informed consent form if they agreed for us to use the results of the evaluation for this study. The procedure for the control group included the standardized cognitive assessment as well as the developmental and behavioral assessments to eliminate the presence of any particular problems (see Instruments below). The project was approved by the research ethics boards of Rivière-des-Prairies Hospital (Project #10-04P) and the University of Sherbrooke (# 2010-111-Parent).

### 2.3. Instruments

#### 2.3.1. ADHD Rating Scale IV (ARS-IV)

The parent and teacher versions of the ARS-IV [70] were used to assess the presence of DSM-IV-TR symptoms of inattention and hyperactivity/impulsivity for ADHD [69] during the biopsychosocial and psychiatric standardized evaluation and rule out the presence of these symptoms in children of the control group. The respondent rated the extent to which the listed behaviors matched those of the child on a Likert scale from 0 to 3 (never or rarely, sometimes, often, very often). This questionnaire has been shown to have high internal consistency (ranging from 0.82 to 0.86 for parents and 0.88 to 0.94 for teachers), test-retest reliability (ranging from 0.78 to 0.86 for parents and 0.88 to 0.90 for teachers), and criterion validity (positive relation with Conners Rating-3 and observation of classroom behavior; and negative relation with academic efficiency score). The ARS-IV remains compatible with the DSM-5 [1] since the symptoms of ADHD have not changed.

#### 2.3.2. Achenbach System of Empirically Based Assessment (ASEBA)

The parent (CBCL/6-18) and teacher (TRF) versions of the ASEBA [71] were used to rule out the presence of internalized and externalized difficulties in the control group. Items are rated on a three-point Likert scale (0 = does not apply, 1 = sometimes true, 2 = often or always true). This instrument has demonstrated acceptable to good test-retest reliability (r = 0.73–0.94 for CBCL and r = 0.60–0.96 for TRF) and internal consistency (α = 0.63–0.97 for CBCL and α = 0.72–0.99 for TRF).

#### 2.3.3. Developmental Questionnaire

This questionnaire was developed by professionals working at the specialized ADHD clinic in Montreal and was used to screen for the presence of mental retardation or neurological problems that would have led to the exclusion of a participant.

#### 2.3.4. The Five-Point Test (FPT)

**Figure 1:**
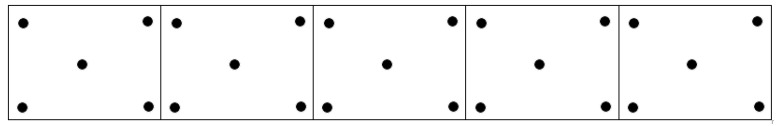
Example of stimuli from the five-point test. The complete test consists of 40 five-point rectangles arranged in 8 rows of 5 rectangles on an 8 ½ × 11 inches sheet.

The FPT [20] requires the child to produce as many novel designs as possible within 5 minutes by connecting at least two of the five dots with a straight line in each square of a matrix. The child was given one or two sheets of paper with 40 squares arranged in an eight-row by five-column matrix (see Figure 1).

Two examples of correct designs were shown. The child could then ask any question before starting. Six scores were used: (a) total number of designs (TD) to assess design production, i.e., generativity; (b) total number of repeated designs (RD) to assess self-regulation, i.e., cognitive flexibility and inhibition (the ability to change a current strategy or to inhibit an automatic, already-produced response); (c) number of correct designs (CD), i.e., TD minus RD, a relative measure of design production; (d) ratio number of RD on TD (RT), to appreciate the importance of repetition relative to design production; (e) ratio number of RD on CD (RC); and (f) total number of strategies (TS) to assess planning. Numerical strategies consisted of adding or subtracting a line from the previous design to create a novel one, and spatial strategies consisted of rotating or mirror flipping the previous design. Construct validity of the FPT is good (0.4 to 0.7). Test-retest reliability is acceptable for CD (0.51) and good for RT (0.78), but remains unknown for the other measures. Design fluency improves with age and is independent of gender and intelligence quotient (IQ) [20,72,73,74,75,76], expect for an effect of gender observed in Chinese children [77] and of IQ in the very superior zone [78,79].

### 2.4. Statistical Analysis

Preliminary analyses were realized. First, one-way analysis of variance (ANOVA) or chi-squared test were performed to determine potential differences between groups in terms of age, gender, or medication. Second, correlation analyses (point-biserial correlations for binary variables: age and medication intake) were carried out between age, gender, medication intake, and FTP scores. Considering the descriptive aim of theses analyses, a significance threshold of 0.05 was considered.

A series of covariance analyses (ANCOVA) were conducted to determine a statistically significant difference between control, ADHD, ADHD-ODD, and ADHD-AD groups on each score of the FPT (TD, RD, CD, RT, RC, and TS), with age as a covariable (given the paucity of FPT age-weighted scores for the age range of our sample). ANCOVA is considered robust to violations of normality and heterogeneity of variance [80]. When a statistically significant difference was observed for a score, pairwise comparisons with Bonferroni adjustment were conducted to specify between which groups the differences lay.

Considering the exploratory aim of the current study, we kept the significance threshold of 0.05. A Bonferroni correction could be used for a more conservative approach. The correction implies a significance threshold of 0.008 (0.05/6). Interpretations are made using the conventional 0.05 threshold, but readers can refer to 0.008 for more conservative results. Analyses were conducted using IBM SPSS (version 23).

## 3. Results

### 3.1. Primary Analyses

A first ANOVA showed that groups did not differ by age in a statistically significant manner, *F*(3,107) = 2.025, *p* = 0.115, η² = 0. 075 (see Table 1). The percentage of participants did not differ by gender between clinical groups, *X*^2^(2, *N* = 77) = 1.49, *p* = 0.475, but did differ between the control and the clinical groups, *X*^2^(3, *N* = 111) = 8.11, *p* = 0.044. The percentage of participants that took medication did not differ between clinical groups, *X*^2^(2, *N* = 77) = 0.20, *p* = 0.903. The three groups with ADHD differed significantly from the control group in terms of symptoms of inattention and hyperactivity (see Table 1). Symptoms, as reported by parents, were at a clinical level (+1 standard deviation). According to the teachers, although the difference is significant, the scores did not reach the clinical threshold, which can be explained by the daily intake of stimulant medication for a large proportion of the sample.

Table 2 shows the correlation coefficients between all variables. Across participants, age showed negligible to low negative correlations with gender (*p* = 0.043, see Table 2) and repetition ratio scores (RT: *p* = 0.003; RC: *p* = 0.002), positive correlations with design production scores in the low (TD: *p* = 0.002) and moderate (CD: *p* < 0.001) range, and a moderate correlation with the strategy score (TS: *p* < 0.001). Gender (0 = female; 1 = male) only showed a low correlation with the repetition scores (RD: *p* = 0.022; RT: *p* = 0.17; RC: *p* = 0.032). Medication intake in the clinical groups (*n* = 77; 0 = unmedicated; 1 = medicated) was not associated to any measure. Given that age was the only variable significantly correlated with the majority of FPT scores, subsequent analyses examining group differences included age as a covariate.

Correlations examining the relationship between the FPT scores indicated that the absolute production score TD was positively associated with all FPT scores, from the low (RT: *p* = 0.001; RC: *p* = 0.007) to moderate (RD: *p* < 0.001; TS: *p* = 0.651), and high (CD: *p <* 0.001) range. The relative production score CD did not correlate with the absolute repetition score RD, but showed a low negative correlation with repetition ratio measures (RT: *p* = 0.019; RC: *p* = 0.007), as well as a high correlation with the strategy score (TS: *p* < 0.001). Finally, the repetition score RD was highly associated with repetition ratio scores (RT: *p* < 0.001; RC: *p* < 0.001), which were very highly associated with each other (*p* < 0.001). The strategy score TS was not associated with any of the repetition scores.

### 3.2. Group Differences

Performance of each group on the FPT together with the results of univariate analyses of covariance are reported in Table 3 (net scores and age-adjusted values).

Results show a statistically significant effect of group on TD, *F*(3,106) = 3.939, *p* = 0.010, η² = 0.100; CD, *F*(3,106) = 2.857, *p* = 0.041, η² = 0. 075; RD, *F*(3,106) = 6.277, *p* = 0.001, η² = 0.151; RT, *F*(3,106) = 5.303, *p* = 0.002, η² = 0. 131, and RC, *F*(3,106) = 4.546, *p* = 0.005, η² = 0. 114. No group effect was observed on TS, *F*(3,106) = 0.714, *p* = 0.546, η² = 0.020.

With regards to the design production measures (TD and CD), multiple pairwise comparisons of adjusted means for age revealed a significant difference on TD between ADHD-AD and ADHD-ODD (*p* = 0.009), and a significant difference on CD between ADHD-AD and ADHD (*p* = 0.030). The ADHD-AD group did not significantly differ on TD from the control (*p* = 0.733) and the ADHD (*p* = 0.097) groups, nor on CD from the control (*p* = 0.132) and ADHD-ODD (*p* = 0.280) groups. No other comparison on TD and CD were significant.

Concerning the repetition measures (RD, RT, and RC), pairwise comparisons revealed a significant difference on RD between ADHD-ODD and the control (*p* < 0.001), ADHD (*p* = 0.008), and ADHD-AD (*p* = 0.008) groups, on RT between ADHD-ODD and the control (*p* = 0.001) and the ADHD (*p* = 0.009) groups, and on RC between ADHD-ODD and the control (*p* = 0.005) and the ADHD (*p* = 0.014) groups. The ADHD-ODD group did not significantly differ from ADHD-AD on RT (*p* = 0.235) and RC (*p* = 0.490). No other comparisons on RD, RT, and RC were significant.

## 4. Discussion

In this study we examined the utility of the design fluency FPT to characterize the cognitive profile of children with ADHD with and without ODD or AD. No previous study on ADHD using design fluency has considered the presence of comorbidities. We predicted that children with comorbid ADHD would offer poorer performance than children with ADHD alone (lower design production and greater repetition) and that the ADHD groups would produce more repeated designs in comparison to the control group.

The results confirm our first hypothesis. The comorbid groups offered poorer performance than the ADHD-alone and control groups. Children with ADHD-ODD made more repeated designs and showed a greater ratio of repeated designs when compared to children with ADHD, ADHD-AD, and non-ADHD children. This suggests lower self-regulation during design production in children with ADHD-ODD, resulting in poor inhibition of already produced responses. Previous studies support a perseveration tendency in children with behavioral disorders [81,82]. The higher number of repeated designs in children with ADHD-ODD may result from specific frontal lobe dysfunction. In their study, Lee et al. [21] observed that adults with frontal lobe damage made more repeated designs on the FPT than those with non-frontal lobe damage. Response perseveration has been specifically associated with dysfunction of the orbitofrontal cortex [83]. Furthermore, lesion of the amygdala has been associated with ODD and behavior problems regardless of the presence of ADHD [60]. This seems to make perfect sense knowing that the amygdala shares neural connections with the frontal and orbitofrontal cortices [84,85]. Thus, the perseveration tendency of children with ADHD-ODD revealed by their performance on the FPT could be linked with orbitofrontal cortex and amygdala dysfunction.

Furthermore, confirming our first hypothesis, children with ADHD-AD produced fewer total designs and correct designs than the ADHD and ADHD-ODD groups, and showed a tendency to produce fewer correct designs than the control group. This suggests that AD impedes generativity in children with ADHD. To our knowledge, no study has compared the design fluency performance of children or adults with ADHD and ADHD-AD. However, studies on individuals with anxiety disorders suggest that the diminution of generativity in the FPT could be due, in part, to frontal and prefrontal cortices dysfunctions. Adults with obsessive-compulsive disorder (OCD) produced fewer correct designs than a control group on the FPT [86], consistent with our results. Furthermore, adults with OCD showed a greater number of repeated designs as well as deficits in other cognitive tasks that require attentional control. The authors suggested that those deficits could stem from specific dysfunctions of the frontomedian cortex. Studies have shown a decreased activity of the dorsolateral prefrontal cortex in individuals with post-traumatic stress disorder [63] and a decreased activity of prefrontal dorsolateral and dorsomedian cortex in individuals with generalized anxiety disorder [64]. This suggests that anxious children may exhibit dysfunction in the neural networks connecting the amygdala and the prefrontal cortex [87]. These dysfunctions may be more specific to AD and explain the lower generativity of children with ADHD-AD in the FPT. Another explanation for the lower generativity of the ADHD-AD group is that anxious children may exhibit an excess of inhibition. The presence of anxiety may increase inhibition in ADHD [88]. Quay [89,90,91] has proposed a theoretical explanation of the ADHD-AD comorbidity based on the neurobiological conceptualization of inhibition of Gray [92], according to which behavior is regulated by the activity of two opposite and interrelated neurobiological systems: the behavioral inhibition system (BIS) and the behavioral activation system (BAS). The activation of one system necessarily deactivates the other. According to Quay’s model, children with ADHD have an underactive BIS, leading to deficits in inhibition and self-regulation, whereas children with AD have an overactive BIS, leading to the behavioral inhibition generally observed in this population. The model therefore predicts that the presence of AD in children with ADHD activates the BIS, which promotes inhibition.

All in all, these results suggest that the dysfunctions associated with ADHD, ODD, and AD are additive. Although these disorders have a common neurobiological basis which may lead to similar cognitive performance, each disorder appears to be associated with specific circuits that are likely to overlap, but also to have a distinct impact on performance, as the results of the present study suggest.

With regards to our second hypothesis, the results do not support the prediction that the ADHD-alone group would underachieve relative to the control group. Both groups provided similar performances on all measures of the FPT. This is in line with previous studies that observed equivalent generativity in children with and without ADHD [33,36]. The absence of a difference between those two groups, however, does not rule out the possibility of an executive deficit in at least a subset of children with ADHD alone. The cognitive heterogeneity observed in youth with ADHD is increasingly recognized [3,8,10] and also supported by the variability observed in the present study (see Table 3), which might level out the results between the ADHD-alone and control groups. Moreover, the absence of a difference between our two groups on the different scores of the FPT does not allow the conclusion that this task is not sensitive to some particularities of ADHD. In Gauthier et al. [36], similar global production performance was observed for both groups, just as in the present study. However, children with ADHD and children of the control group showed distinct production patterns during the task, suggesting that process measures may be more sensitive than global performance scores in capturing cognitive processing, at least in regards to the total number of designs.

There might be other explanations for the lack of a difference between our control and ADHD-alone groups, which could also account for the cognitive heterogeneity observed in ADHD. First, some authors suggested the possibility that executive dysfunction in ADHD may be partially due to an impairment of automatic processing (e.g., [93,94,95]). Basic processes that are not well automatized may result in executive deficits [94], for example a high cognitive load ending up competing for limited resources used by executive functions. A second possible explanation is that ADHD may not equate with interchangeable multiple specific deficits as proposed by multipath theories [96], but rather stem from a more general cognitive coordination impairment (e.g., [97]). Dynamic coordination is increasingly recognized as an important adaptive characteristic of the brain and mind [98]. Cognitive coordination may be conceived as the ability to dynamically organize and optimally deploy energy resources to support cognitive systems that are needed for performing a task and achieving its goals. Design fluency is a complex task that involves pursuing multiple goals (e.g., produce designs, avoid repetition, and make straight and dot-to-dot lines). The task must therefore be facilitated by coordinating multiple cognitive processes (generativity, self-regulation, planning, working memory, motor control, etc.) that are distributed in multiple neural networks. A deficit in cognitive coordination may not manifest itself in group comparisons, as it could impede performance on one measure at some point in time and on another measure at some other time, thus at the end averaging out the data. For example, a child with a cognitive coordination deficit may focus on generating designs while simultaneously having difficulty monitoring performance and do the opposite one moment later. This would result in a production pattern where a high amount of designs and repetitions is followed by low error and generativity. Such alternating design production pattern during the FPT is exactly what characterized children with ADHD in Gauthier et al. [36]. In the present study, children with ADHD-ODD produced a high number of designs but also a greater number of errors, whereas children with ADHD-AD produced fewer errors, but also fewer designs. This could be the additive contribution of ADHD’s cognitive coordination deficit to ODD- and AD- specific impairments. In support of this explanation, recent evidence suggests that ADHD is associated with an N-methyl-D-aspartate (NMDA) glutamate receptor dysfunction [99], which is also held responsible for the cognitive coordination deficit observed in autism spectrum disorder and schizophrenia [100,101].

Finally, the results of the correlational analyses are interesting from a clinical point of view. They are congruent with previous studies showing an effect of age, but not of gender, on productivity in design fluency [20,72,73,74,75,76]. The number of correct designs increased with age, in both the control and clinical groups. Our results further suggest that the use of strategy increases with age and that the ratio of repeated designs decreases. Thus, younger children showed lower performance on production, strategy, and repetition ratio scores, congruent with research evidence of child executive function development at this age (e.g., [102]) and with the potential utility of the FPT for characterizing such development. Interestingly, strategy was positively associated with productivity, but showed no effect on repetition scores. The use of strategy therefore seems to promote generativity, but not reduce repeated designs, as previously proposed [19]. Finally, we conducted a point-biserial correlation to determine if medication intake was associated with better scores on the FPT, as ADHD medication may have a long-term effect on the brain [103,104,105]. No significant association was observed (we conducted further univariate analyses (non-reported) to rule out (and which showed the absence of) a possible lasting effect of daily medication on the performances of children with ADHD on the FPT).

### Strengths, Limitations, and Implications for Future Research

Although a rigorous, standardized, and comprehensive diagnostic procedure was used, we did not control for the severity of symptoms related to ADHD, ODD, and AD, and the ADHD-AD group may have included different anxiety disorders that may have masked their effect on the performance. Future research should compare groups of children with different anxiety disorders while controlling for the severity of anxiety (and other disorders) symptoms. Furthermore, the small sample size may explain the absence of a significant difference in generativity between the ADHD-AD and the control groups. Finally, adding process measures to global performance scores may help better characterize the cognitive profiles of children with ADHD with and without comorbidities, and identify differences between groups (see [36]).

## 5. Conclusions

In this study, children with comorbid ADHD performed less well than children with or without ADHD on the FPT, a measure of executive function that recruits and requires the coordination of multiple cognitive processes. Compared to other groups, children with ADHD-ODD made more repeated designs, which may reflect poorer self-regulation, while children with ADHD-AD produced fewer designs, showing lower generativity that may reflect an excess of inhibition. The performance of comorbid ADHD groups on the FPT may be associated with neurobiological dysfunctions that are specific to ODD and AD. Neural networks connecting the amygdala to the frontal and prefrontal regions are suspected. A deficit in cognitive coordination may also contribute to the poorer performance of the ADHD-ODD and ADHD-AD groups. In conclusion, the results of the present study suggest that the cognitive impairments associated with ADHD, ODD, and AD are additive. Although further research is needed to confirm this, it still remains that the presence of comorbidities is associated with greater functional impacts that are not always corroborated by the child’s performance in the context of neuropsychological studies or clinical assessment. Considering the high frequency of these comorbidities, it appears important to develop tools that are more sensitive to the cognitive deficits that may characterize children with ADHD, with or without comorbid disorders. This would help to understand the impact of comorbid disorders on everyday functioning and guide specific interventions. The present study suggests that design fluency could be helpful in this regard.

## Figures and Tables

**Table 1:** Participant demographics by group.

	Control	ADHD	ADHD-ODD	ADHD-AD	
	(*n* = 34)	(*n* = 39)	(*n* = 20)	(*n* = 18)	Comparisons
Age: mean (SD)	10.03 (1.06)	9.63 (0.86)	9.73 (0.99)	9.38 (1.02)	ns
Gender: male *n* (%)	16 (47.06)	26 (66.67)	16 (80.00)	14 (77.78)	Control < Clinical
Medication: active *n* (%)	n/a	21 (53.85)	12 (60.00)	10 (55.56)	ns
ADHD symptoms (parent): mean (SD)	32.44 (20.35)	92.08 (11.55)	94.70 (10.67)	88.50 (12.23)	Control < Clinical
ADHD symptoms (teacher): mean (SD)	24.03 (23.06)	70.86 (20.17)	72.60 (24.31)	62.06 (24.28)	Control < Clinical

SD: standard deviation; n/a = not applicable; ns = not significant; ADHD = attention deficit/hyperactivity disorder; ADHD-ODD = ADHD with associated oppositional defiant disorder; ADHD-AD = ADHD with associated anxiety disorder. Medication is essentially long action psychostimulants. ADHD symptoms (in percentile) measured with ADHD Rating Scale IV (ARS-IV).

**Table 2:** Pearson correlation coefficients for age, gender, medication, and FPT scores.

	1	2 ^a^	3 ^b^	4	5	6	7	8	9
1. Age	–								
2. Gender	−0.192 *	–							
3. Medication	0.036	−0.075	–						
4. TD	0.290 **	0.085	0.169	–					
5. CD	0.467 ***	−0.041	0.143	0.844 ***	–				
6. RD	−0.144	0.217 *	0.116	0.620 ***	0.102	–			
7. RT	−0.282 **	0.227 *	0.057	0.304 **	−0.223 *	0.888 ***	–		
8. RC	−0.290 **	0.203 *	0.014	0.256 **	−0.254 **	0.846 ***	0.968 ***	–	
9. TS	0.482 ***	−0.126	0.091	0.651 ***	0.746 ***	0.117	−0.063	−0.062	–

^a^ Point-biserial correlation (0 = female; 1 = male); ^b^ Point-biserial correlation for clinical groups only (0 = unmedicated; 1 = medicated). FPT = five-point test; TD = total designs; CD = correct designs; RD = repeated designs; RT = repeated on total designs; RC = repeated on correct designs; TS = total number of strategy designs. * *p* < 0.05; ** *p* < 0.01; *** *p* < 0.001.

**Table 3:** Performance and group differences across the FPT measures.

	Mean (SD) and Adjusted Mean (SE) for Age * for Each Group						
	Control	ADHD	ADHD-ODD	ADHD-AD	*F*	*p*	η²
FPT Measure							
TD	34.53 (9.45)	35.77 (11.56)	40.20 (15.17)	27.22 (9.87)			
	33.52 (1.92)	36.11 (1.77)	40.20 (2.47) ^a^	28.38 (2.63) ^a^	3.94	0.010	0.100
CD	31.12 (8.84)	30.46 (8.74)	29.55 (9.46)	22.72 (9.85)			
	29.85 (1.42)	30.89 (1.31) ^a^	29.56 (1.83)	24.18 (1.95) ^a^	2.86	0.41	0.075
RD	3.41 (3.93)	5.31 (5.97)	10.65 (9.34)	4.50 (4.59)			
	3.46 (1.07) ^c^	5.27 (0.97) ^a^	10.65 (1.35) ^a^	4.01 (1.50) ^a^	6.28	0.001	0.151
TS	11.06 (8.35)	11.26 (7.41)	9.25 (4.93)	8.83 (8.49)			
	9.92 (1.16)	11.64 (1.06)	9.26 (1.48)	10.14 (1.58)	0.71	0.546	0.020
RT	9.39 (9.43)	13.18 (11.83)	24.05 (16.23)	16.75 (16.42)			
	10.50 (2.20) ^f^	12.80 (2.00) ^a^	24.00 (2.80) ^a^	15.50 (3.00)	5.30	0.002	0.131
RC	11.97 (16.20)	17.86 (20.71)	38.46 (34.09)	26.73 (37.22)			
	14.20 (4.30) ^a^	17.10 (4.00) ^a^	38.50 (5.50) ^a^	24.20 (5.90)	4.55	0.005	0.114

SD: standard deviation; SE: standard error; *, The covariate in the model is evaluated using the value: age = 9.73. FPT = five-point test; TD = total designs; CD = correct designs; RD = repeated designs; TS = strategy designs; RT = repeated on total designs; RC = repeated on correct designs. Superscript a, c, f indicate statistically significant differences between groups after adjustment for multiple comparisons (Bonferroni).
